# Prevalence of Dental Caries in Adults Scheduled for Hematopoietic Stem Cell Transplantation: A Multicenter Study in a Polish Population

**DOI:** 10.3390/cancers18091383

**Published:** 2026-04-27

**Authors:** Agnieszka Bogusławska-Kapała, Andrzej Miskiewicz, Barbara Kochańska, Aida Kusiak, Barbara A. Jereczek-Fossa, Agnieszka Banatkiewicz, Joanna Gordon-Piotrowska, Izabela Strużycka, Bartłomiej Górski, Aniela Brodzikowska

**Affiliations:** 1 Department of Comprehensive Dental Care, Medical University of Warsaw, 02-091 Warsaw, Poland; agnieszka.boguslawska-kapala@wum.edu.pl (A.B.-K.); agnieszka.banatkiewicz@wum.edu.pl (A.B.); joanna.gordon-piotrowska@wum.edu.pl (J.G.-P.); izabela.struzycka@wum.edu.pl (I.S.); 2 Department of Periodontology and Oral Diseases, Medical University of Warsaw, 02-091 Warsaw, Poland; andrzej.miskiewicz@wum.edu.pl (A.M.); bartlomiej.gorski@wum.edu.pl (B.G.); 3 Department of Conservative Dentistry, Medical University of Gdańsk, 80-210 Gdańsk, Poland; barbara.kochanska@gumed.edu.pl; 4 Department of Periodontology and Oral Mucosa Diseases, Medical University of Gdańsk, 80-210 Gdańsk, Poland; aida.kusiak@gumed.edu.pl; 5 Department of Oncology and Hemato-Oncology, University of Milan, 20122 Milan, Italy; barbara.jereczek@ieo.it; 6 Department of Radiation Oncology, IEO European Institute of Oncology IRCCS, 20141 Milan, Italy; 7 Department of Conservative Dentistry, Medical University of Warsaw, 02-091 Warsaw, Poland

**Keywords:** oncological therapy, hematopoietic stem cell transplantation, dental caries, frequency, risk factors, oral health protocols

## Abstract

**Background**: Preparing patients for oncological therapy requires the elimination of foci of infection in accordance with Multinational Association of Supportive Care in Cancer/International Society of Oral Oncology (MASCC/ISOO) guidelines. Complications of dental caries, such as abscesses, can lead to sepsis. In Poland, the pre-oncologic dental treatment of patients with hematopoietic disorders does not yet meet MASCC/ISOO standards; however, an inventory of the current status of dental care for these patients is ongoing. The aim of this study was to investigate the frequency of dental caries and to define the local caries risk factors in adult patients prior to Hematopoietic Stem Cell Transplantation (HSCT) as a part of anticancer therapy. Additionally, the time available for dental treatment was assessed. **Methods**: A total of 302 patients were examined. Dental status was determined based on the number of decayed, missing and filled teeth and on treatment needs. Local caries risk factors, such as poor dietary habits, insufficient oral hygiene, and symptoms of reduced salivary flow, were examined. The diet was assessed using a questionnaire; tooth cleaning efficiency was assessed as a percentage of dental surfaces with biofilm. Symptoms of reduced salivary flow were determined by subjective and clinical signs of low salivary secretion. **Results**: Active (progressive) dental caries was diagnosed in 85.2% of patients. Insufficient oral hygiene had been found in 71.52% of those examined. Symptoms of hyposalivation were present in 85% of patients. In 31% of cases, time available for dental treatment prior to HSCT was too short. **Conclusions**: Tooth decay, the presence of caries risk factors, and insufficient time for oral treatment in patients submitted to HSCT represent a serious clinical problem. There is a clear need to establish comprehensive oral health protocols aimed at providing patients with appropriate, urgent dental care. Furthermore, coordination between oncologists and dentists in Poland must be improved. Currently patients are rarely referred to a dentist before starting anticancer therapy; those scheduled to hematopoietic stem cell transplantation, usually see a dentist too late.

## Simple Summary

This study evaluated the dental status of adults aged 18–71 years who were eligible for Hematopoietic Stem Cell Transplantation (HSCT) as part of oncologic treatment at three Polish transplant centers. The aim was to investigate the prevalence and severity of dental caries and to assess local risk factors, specifically oral hygiene and hyposalivation. Additionally, the study evaluated the time available for dental treatment prior to transplantation. Dental caries, insufficient oral hygiene, and the subjective and indirect symptoms of reduced salivary flow were found in the majority of patients. In one-third of the cases, the time available to perform necessary dental procedures before HSCT was insufficient. These findings suggest that the oncological and dental healthcare systems in Poland urgently require better integration.

## 1. Background

According to International Agency for Research on Cancer (IARC), there were an estimated 20 million new cancer cases worldwide in 2022. Moreover, in the next two decades, the number of people with new diagnosed cancer is expected to rise approximately 50% [1].

Hematopoietic stem cell transplantation (HSCT) is now a common treatment for malignant solid and hematological tumors [2]. The number of patients undergoing HSCT is steadily increasing; for example, 48,512 transplants were performed in Europe in 2019 [2].

Despite advances in therapy, anticancer treatment still carries a high risk of various complications, which often localize in the oral cavity [3]. Of particular concern are oral diseases that can be a source of systemic infection threatening the life or health of patients [3]. These primarily include periodontopathies and dental caries and their resulting complications [4,5].

According to the report by Organization of Caries Research (ORCA) and International Association for Dental Research (IADR), dental caries is “a biofilm-mediated, diet modulated, multifactorial, non-communicable, dynamic disease resulting in net mineral loss of dental hard tissues” [6]. Four principal factors play a major role in the etiology of dental caries: host properties (including reduced saliva secretion and the micro- and macrostructure of teeth), cariogenic biofilm, a diet rich in simple sugars, and the duration/timing of the these factors [6].

Saliva is a complex fluid fundamental to maintaining oral homeostasis. Its functions include, among others, the mechanical cleaning of teeth, the antibacterial action of salivary components, the neutralization of acids produced by bacteria, and the remineralization of dental hard tissues [7]. Inadequate saliva secretion can lead to the development of numerous oral diseases, including the rapid progression of dental caries [7].

Frequent consumption of simple sugars and inappropriate oral hygiene represent other high-risk factors for dental caries. They contribute substantially to the formation of cariogenic biofilms, which increase the risk of developing carious lesions [8]. In the presence of fermentable carbohydrates, the ecological balance on the tooth surface is disturbed [8]. By metabolizing the supplied carbohydrates, bacteria produce large amounts of acid, which ultimately leads to a shift toward demineralization of dental hard tissues and the formation of a carious lesion [9].

Untreated dental caries may lead to inflammatory changes in the pulp, periodontium, and periapical tissues [10]. These conditions are not only a cause of pain but, more importantly, carry the risk of spreading infection to surrounding tissues and a higher risk of blood stream infections [10,11].

Moreover, untreated deep caries requires the implementation of invasive procedures, such as root canal treatment and extractions. During these procedures, pathogenic plaque can enter the circulation [10]. Transient bacteremia originating from carious lesions can also occur during daily activities, such as tooth brushing or even chewing [11]. The loss of teeth due to dental caries or its complications often results in the development of masticatory dysfunction. The loss of even a single tooth can, under some circumstances, lead to changes in occlusion and deterioration of chewing function [12]. Consequently, the digestive process may be impaired, which can negatively affect the healing process in the post-HSCT period [13]. Recent studies have shown that tooth loss is associated with the incidence of cardiovascular diseases, such as myocardial infarction, heart failure, and ischemic stroke [14]. Moreover, tooth loss often compromises aesthetics and speech function, thereby reducing the patient’s quality of life [15].

Based on the current knowledge, the Multinational Association of Supportive Care in Cancer/International Society of Oral Oncology (MASCC/ISOO) has developed standards for treating patients before HSCT [3]. These standards are constantly updated. In Poland, the pre-oncologic dental treatment of patients with hematopoietic disorders does not yet meet MASCC/ISOO guidelines; however, an inventory of the current status of dental care for these patients is ongoing.

Despite the scale of the problem, epidemiological studies on the prevalence of dental caries in adult patients qualifying for HSCT as a part of oncological treatment are scarce [16,17]. None of the existing studies covers data from Poland, where the frequency of dental caries in the general population is high [18]. Furthermore, there are also no reports focusing on factors affecting caries development that may be specific to patients prior to HSCT. Additionally, several papers have highlighted the significant issue of an insufficient time interval for dental treatment between the initial dental referral and the start of HSCT [17]. An analysis of these problems could be instrumental in developing a detailed protocol for preventive and therapeutic treatments of tooth decay in patients being prepared for oncological treatment or transplantation in Poland.

The aim of this study was to analyze the frequency of tooth decay and the treatment needs associated with dental caries in the Polish population of adults preceding transplantation. The prevalence of select caries risk factors was also assessed, including the frequent consumption of simple carbohydrates, inadequate oral hygiene, and salivary disorders. Additionally, treatment needs were analyzed in terms of the time remaining before the start of transplantation therapy.

## 2. Materials and Methods

The study included 302 participants, 137 females (45.3%) and 165 males (54.7%), aged from 18 to 71 years (M = 46.1; SD = 14.5).

Inclusion criteria: the study included patients undergoing preparation for alloHSCT (254 participants, 84%) and patients undergoing preparation for autoHSCT (48 participants, 16%), who consented to participate in the study.

Exclusion criteria: patients who were edentulous and those who did not consent to participate in the study.

The diagnoses are presented in Table 1.

The average duration of treatment of the underlying disease (i.e., chemotherapy) was M = 14 months (SD = 17.2).

The study was conducted from 2017 to 2022 in three leading Polish transplant centers: Department of Hematology, Transplantology and Internal Diseases—Medical University of Warsaw, Institute of Hematology and Blood Transfusion in Warsaw, and Department of Haematology and Transplantology—Medical University of Gdańsk. Dental examinations were conducted in Department of Comprehensive Dentistry—Medical University of Warsaw and in Department of Conservative Dentistry—Medical University of Gdańsk. All patients were referred to the dental clinic for oral evaluation as part of pre-transplant procedures.

Approval for the study was obtained from the Bioethics Committee of Medical University of Warsaw, Poland (protocol code KB/54/A/2017). The ethics approval date was 4 July 2017. This study was conducted in line with the principles of the Declaration of Helsinki. Every patient gave informed consent to participate in the study.

The study used the Epidemiological Survey Card model, according to the World Health Organization (WHO) Oral Health Assessment Forms (Oral Health Surveys. Basic methods, 4th Edition, Geneva 1997, and 5th Edition, Geneva 2013) [19]. Only one investigator participated in the study.

In the patients’ general history, data including the diagnosis of the underlying disease, its previous course with treatment, the planned date of the commencement of the transplantation procedure, other diseases past or concomitant, and medications taken were collected.

The dental interview included, inter alia, questions related to the frequency of consumption of simple sugars outside of main meals (sweets, confectionery products, juices, and sweetened beverages) and hygiene habits, such as the frequency of tooth brushing.

Clinical evaluation of the oral cavity was conducted in a dental office setting, using sight and touch, under artificial light from the dental chair. Each patient had an orthopantomographic X-ray and intraoral X-rays (dental status) taken.

Decreased salivary secretion was assessed based on the presence or absence of subjective feelings of dry mouth in the history and by the degree of oral mucosa hydration as found by clinical examination.

A questionnaire and a numerical scale—similar to the Numerical Rating Scale (NRS) used in pain assessment—were employed to estimate the daily discomfort experienced by patients due to xerostomia. The questionnaire included the following questions from the study by Sreebny and Valdini [20]:

- Does your mouth usually feel dry?
- Do you get out of bed at night to drink fluids?
- Does your mouth usually become dry when you speak?

We also added an additional question:

- Do you have difficulty eating dry foods?

The severity of the subjective feeling of dry mouth was rated by patients on a scale of 0–10, where 0 meant no subjective feeling of dryness and 10 meant a severe feeling of dry mouth [21].

The degree of mucosal hydration was assessed by sight and touch, then represented on a three-point scale:

- 1° hydration—proper hydration of the mucosa.
- 2° hydration—insufficient hydration of the mucosa—sticking of an intraoral mirror or glove to the buccal mucosa or tongue.
- 3° hydration—symptoms of dry mucosa [22,23]: frothy saliva; no saliva pooling in floor of mouth; no saliva collection on the mirror placed over both parotid gland openings; loss of papillae of the tongue dorsum; altered/smooth gingival architecture; glassy appearance to the oral mucosa (especially the palate); deeply fissured tongue; cervical caries (more than two teeth); and mucosal debris on palate or gingiva [23].

The oral mucosa was always examined at the start of the clinical trial so as not to distort the clinical picture, i.e., to avoid mucosal drying caused by the patient keeping their mouth open for a long time. To enhance the reliability of the study, the questionnaire and the assessment of clinical symptoms of dryness were repeated at the start of each dental visit related to procedures necessary prior to HSCT. In assessing the prevalence of caries, the frequency of active caries and the intensity of caries were considered. According to ORCA and International Caries Detection and Assessment System (ICDAS) recommendations, active versus arrested caries were thoroughly diagnosed during the clinical examination [24]. This distinction directly influenced the dental therapy planning.

Caries activity (both primary and secondary lesions) was assessed using visual–tactile methods, supplemented by bitewing radiography. The following clinical features of the lesions were taken into account: color, translucency, hardness on probing, surface texture changes, and the presence of dental plaque. Color was considered an additional factor [25]. The hardness of the caries lesion was assessed with a rounded dental explorer to avoid damaging the tooth surface [25].

A lesion was classified as active when it was whitish or yellowish, lacked shine, felt rough, soft, or leathery on probing, and was covered with plaque. Following the criteria of Ekstrand and Nyvad et al., non-cleansable cavitated lesions were considered active unless clear signs of inactivity were present. Non-cavitated lesions with an uncertain activity status were also regarded as active [26,27]. The frequency of active caries was expressed as the percentage of subjects with Decayed Teeth (DT) score > 0. The intensity of caries in individual patients was assessed by the Decayed, Missing, and Filled Teeth (DMFT) score, which is the sum of its components (DT + MT + FT) [28]. The DMFT Index (which is the average DMFT per person) was calculated for the entire population. The Significant Caries Index (SiC) (the average DMFT in the group of 33% of people with the highest DMFT values) was also calculated [29].

To rate the caries treatment needs, the Treatment Needs Index (TNI), described by WHO, was used [30]. TNI calculates the relationship between untreated decayed teeth and teeth treated with restorations or extractions (due to caries). To evaluate the efficacy of restorative caries treatment, the treatment index was calculated according to the FT/(DT + FT) formula [29].

The Approximal Plaque Index (API) according to Lange was used to evaluate the efficacy of elimination of oral biofilm by hygiene interventions [31].

To address the research questions, statistical analysis of the obtained database was conducted, using IBM SPSS Statistics v.25 software (IBM, New York, NY, USA). The basic descriptive statistics were calculated. Moreover, the Kolmogorov–Smirnov test, Student’s *t*-test, Mann–Whitney’s test, Kruskal–Wallis non-parametric ANOVA test, Pearson’s correlation coefficient (r), Spearman’s rank correlation and a logistic regression analysis with progressive Wald selection were used. The alpha value threshold used was 0.05 (*p* < 0.05).

## 3. Results

The descriptive statistics are shown in Table 2, Table 3 and Table 4. The Kolmogorov–Smirnov test was statistically significant, meaning that the distribution of the scores was significantly different from the normal distribution. However, both skewness and kurtosis were smaller than the absolute value of 2, so it was proper to use parametric tests.

### 3.1. Dental Caries Prevalence and Severity; Caries Treatment Needs

The frequency of active caries in the studied group amounted to 85.1% (*N* = 257). Only 1 out of 302 persons was completely free from tooth decay or its effects (DMFT = 0).

Indices regarding dental caries severity and treatment needs are shown in Table 2. On average, over five teeth were affected by active caries. Particular attention was drawn to the high values regarding the SiC Index.

**Table 2:** Distribution of the tooth indices in the patient group before HSCT.

Indices	M	Me	SD	Min	Max
Remaining teeth [*n*]	23.6	26	7.6	2	32
Decayed teeth (DT) [*n*]	5.2	4	4.6	0	24
Missing teeth (MT) [*n*]	7.5	5	7.9	0	28
Filled teeth (FT) [*n*]	6.5	6	5.3	0	22
DMFT Index [*n*]	19	20	8	0	32
SiC Index [*n*]	27.5	27	2.5	24	32
FT/(DT + FT) formula	0.5	0.6	0.3	0	1
TNI	0.9	0.4	1.7	0	11

DMFT Index = Decayed (DT) + Missing (MT) + Filled (FT) Teeth Index; SiC Index—Significant Caries Index; TNI—Treatment Need Index; M—mean; Me—median; SD—standard deviation; Min—minimum value; Max—maximum value.

### 3.2. Time Remaining Before the Start of the HSCT Procedure

Table 3 shows the data concerning the time remaining until the start of the transplant procedure at the patient’s first dental visit. In 11 people, the date of transplantation has not been determined yet. In the remaining study group (*N* = 291), patients were referred to the dentist about 3 weeks prior to the planned date of transplantation. In 42 persons DT = 0. Out of 249 patients with active caries (DT > 0), in 78 cases, the time remaining until the transplantation ranged from three days to two weeks. At this time, on average, over six teeth needed the conservative treatment, and this required a median of two visits (barring the initial dental referral) (Table 3).

**Table 3:** Time remaining until HSCT, the number of teeth requiring treatment for active caries (DT > 0), and the number of necessary visits between the initial dental referral and transplantation.

Time Between Initial Dental Referral and HSCT	Persons with DT Index > 0	Number of Teeth Requiring Treatment for Caries (DT Index > 0)					Number of Visits Between Initial Dental Referral and HSCT				
Weeks	*N* (%)	M	Me	SD	Min	Max	M	Me	SD	Min	Max
0.5–2	78 (31.33%)	6.7	6	4.5	1	21	2.6	2	1.5	1	8
3–6	143 (57.42%)	5.8	5	4.4	1	24	2.4	2	1.7	1	8
8–12	28 (11.24%)	6.8	6.5	4.1	2	16	2.9	2.5	1.9	1	8
0.5–12 M = 3.7 Me = 4 SD = 2.4	249 (82.45%)	6.2	5	4.4	1	24	2.5	2	1.7	1	8

M—mean; Me—median; SD—standard deviation; Min—minimum value; Max—maximum value.

### 3.3. Caries Risk Factors

Analysis of data concerning oral hygiene habits in patients prepared for HSCT showed great negligence. Average API values confirmed that the patients’ oral hygiene was poor (Table 4).

**Table 4:** Selected caries risk factors: poor oral hygiene (API) and symptoms of reduced salivary flow in patients before HSCT (*N* = 302).

Local Factors		M	Me	SD	Min	Max
Oral hygiene (API)		56	56.3	25.1	0.3	100
Self-perceived degree of dry mouth * (*N* = 80)		1.4	0	2.5	0	10
Oral mucosa hydration	1°—proper hydration	195 (64.57%)				
	2°—mucous membrane moisturized insufficiently	99 (32.78%)				
	3°—dryness	8 (2.65%)				

* Self-perceived degree of dry mouth: 0—lack of dry mouth sensation; 10—maximum dry mouth sensation; API—Approximal Plaque Index; M—mean; Me—median; SD—standard deviation; Min—minimum value; Max—maximum value.

To investigate which variables predict active caries (teeth with caries), a logistic regression analysis with progressive Wald selection was performed. Two statistically significant predictors were included in the model: API level and oral mucosal hydration, χ^2^(2) = 22.70; *p* < 0.001; *R*^2^*_Cox-Snell_* = 0.07; *R*^2^*_Negelkerke_* = 0.10. The presence of active caries could be predicted by a higher API value and poorer oral hydration. These data allowed for 90.2% correct classification of the presence of active caries (175 correct classifications and 19 incorrect classifications) and 27.5% of the absence of active caries (27 correct classifications and 82 incorrect classifications). The remaining variables were not included in the model as they were not statistically significant predictors. The results are presented in Table 5.

In 71.52% of examined persons oral hygiene needed improvement. Up to 30% of patients either did not brush their teeth or did so only once a day (Table 6).

Additionally, symptoms of insufficient salivary secretion were found in 85% of the patients before HSCT.

Among 302 individuals, 73 (24.17%) admitted to consuming simple sugars outside of main meals.

We found a weak positive correlation between the decayed teeth number (DT Index) and the self-perceived degree of dry mouth (r = 0.27; *p* < 0.001) as well as the hydration of the mucosa level (rs = 0.28; *p* < 0.001). The higher the decayed teeth number, the higher the intensity of the symptoms was.

Furthermore, the Pearson’s correlation coefficient (r) was also calculated to verify the correlation between the API and the DMFT values. A statistically significant, moderately strong correlation was found; API value was positively correlated with DMFT (r = 0.31; *p* < 0.001).

In the analysis that followed, the Kruskal–Wallis non-parametric ANOVA test was performed to verify the correlation between the patients’ teeth brushing frequency and the DMFT, SiC, and API. Two statistically significant results were found: DMFT and API. All results are presented in Table 6.

The Dunn–Sidak post hoc test was performed for all three indices. As for the DMFT Index, two statistically significant differences were found. Participants who brushed their teeth two times a day got lower DMFT Index values than participants who brushed their teeth once a day (*p* < 0.001) and then participants who sporadically or never brushed their teeth (*p* < 0.001). Additionally, statistically significant differences for the API were found. Participants who brushed their teeth two times a day got lower API values than participants who brushed their teeth once a day (*p* < 0.001) and then participants who sporadically or never brushed their teeth (*p* < 0.001). Participants who brushed their teeth once per day also got lower API levels than participants who sporadically or never brushed their teeth (*p* = 0.025).

The impact summary of sugar consumption as a grouping variable was verified by U Mann–Whitney and W Wilcoxon tests. In the analysis we found a strong asymptotic two-sided value between the decayed teeth number (DT > 0) and frequent consumption of carbohydrates (*p* = 0.010).

## 4. Discussion

**Figure 1:**
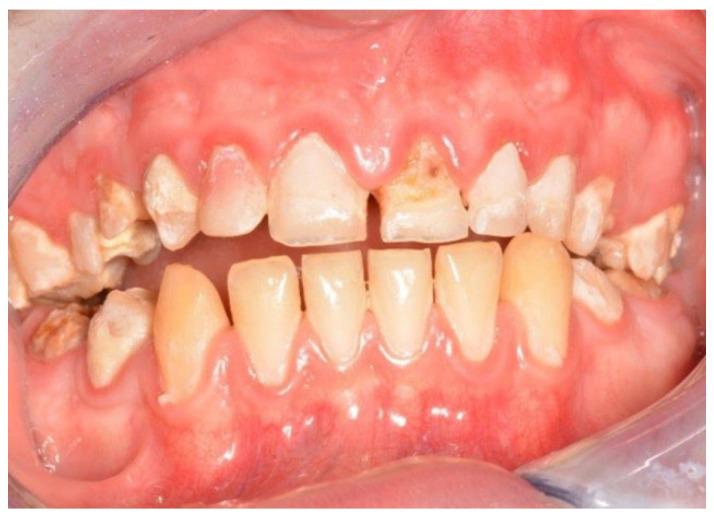
21-year-old woman, 4 weeks to alloHSCT.

**Figure 2:**
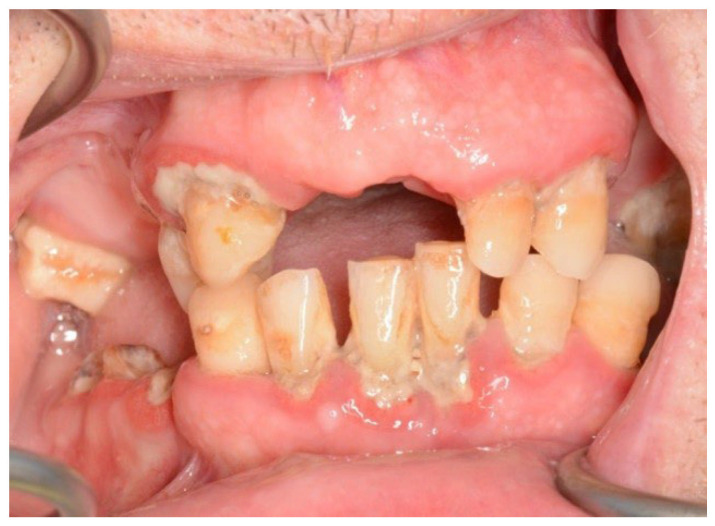
23-year-old man, 1 week to alloHSCT.

**Figure 3:**
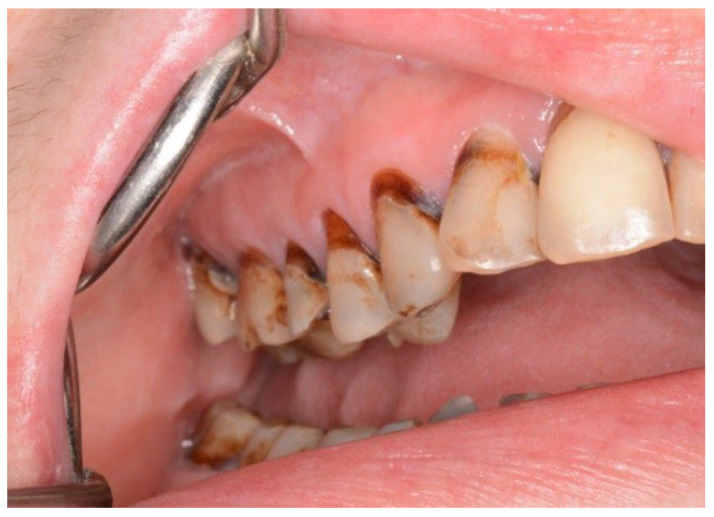
45-year-old woman, first dental visit 1 year after alloHSCT, cGvHD, assessing of salivary rate impossible due to little amount of sticky saliva.

**Figure 4:**
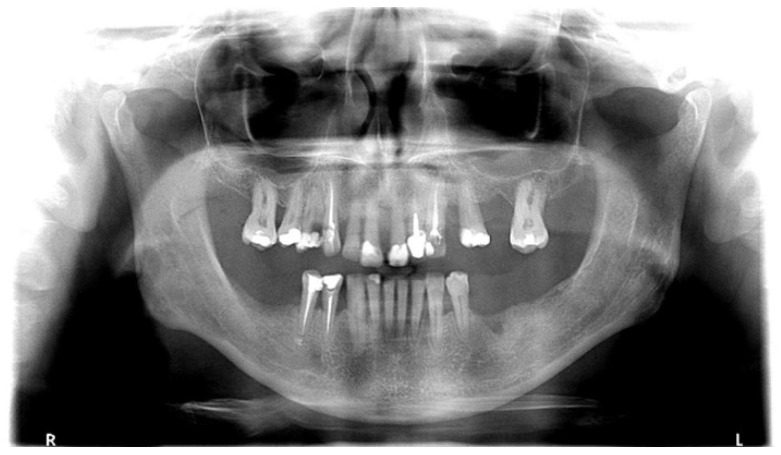
63-year-old man, 6 weeks to alloHSCT.

**Figure 5:**
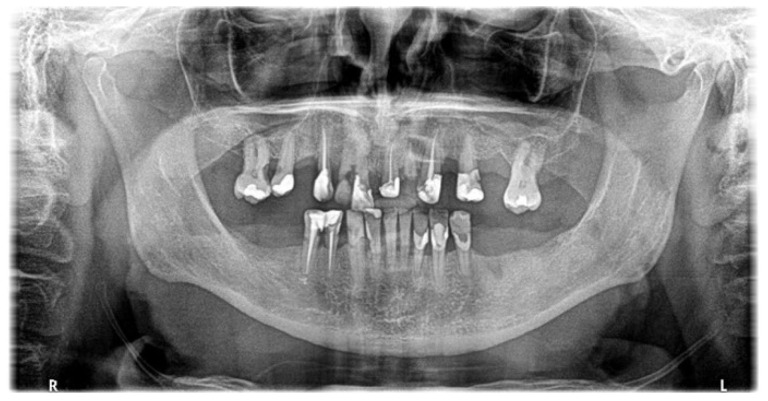
The same patient (Figure 4), first dental visit 18 months after alloHSCT, cGvHD, lack of saliva.

In both the early and late stages following HSCT, patients are at high risk of complications [3]. Among these, infections often originate from the oral cavity [11]. Additionally, patients frequently experience poor general well-being and oral discomfort, such as xerostomia or pain [3]. These complaints often lead patients to abandon regular oral care and routine dental visits (Figure 1 and Figure 2). Under such conditions, tooth decay can develop rapidly, leading to complications, particularly systemic infections (Figure 3, Figure 4 and Figure 5) [32,33,34]. Therefore, it is crucial to treat caries and its complications before initiating the transplantation procedure. Furthermore, it is essential to identify factors predisposing patients to caries prior to HSCT and implement individualized prevention programs based on MASCC/ISOO guidelines [3]. In Poland, the pre-oncologic dental treatment of patients with hematopoietic disorders does not yet meet these standards.

The data presented in our study confirm a high need for caries treatment and prevention among the Polish population prior to HSCT. In the study group, the prevalence of active tooth decay was nearly 85%, which is substantially higher than the results reported in several other European countries [35]. The mean Decayed Teeth (DT) index in these subjects was also high. Notably, high caries intensity was observed in the top 33% of participants with the highest DMFT scores (SiC Index). Compared to oral health monitoring data for the general Polish population, patients qualifying for HSCT exhibited higher caries intensity [18]. This was particularly evident among young adults (18–34 years) and those aged 35–44. For instance, patients awaiting HSCT aged 18–34 had, on average, 5.6 teeth with active caries, whereas 18-year-olds in the general population had a considerably lower DT index of 3 [18].

Due to a lack of existing reports, these findings could not be compared with data on HSCT patients from other Central-Eastern European populations. Epidemiological studies on caries prevalence in adults before HSCT in other regions are also scarce [5,17]. Similar DMFT values (M = 17) were reported by Hansen et al. in a group of 350 patients at Memorial Sloan Kettering Cancer Center (NY, USA), though data concerning active caries (DT > 0) were not provided [5]. Durey et al., evaluating 116 patients in Edinburgh (UK) prior to HSCT, found a significantly lower prevalence of active caries (52%) compared to our study group [17].

Another issue noted was the low coverage of prior dental treatment needs in HSCT candidates compared to the general population. In Poland, the restorative treatment index for the general population (aged 35–44) averages 0.7 (SD = 0.3) [18]. The low treatment coverage in the HSCT group may be due to the long duration of the underlying disease (M = 14 months; SD = 17), during which the patient’s oral health care and dental visit frequency may have been impeded by deteriorating health. No analogous data for pre-HSCT patients were found in the available literature.

**Figure 6:**
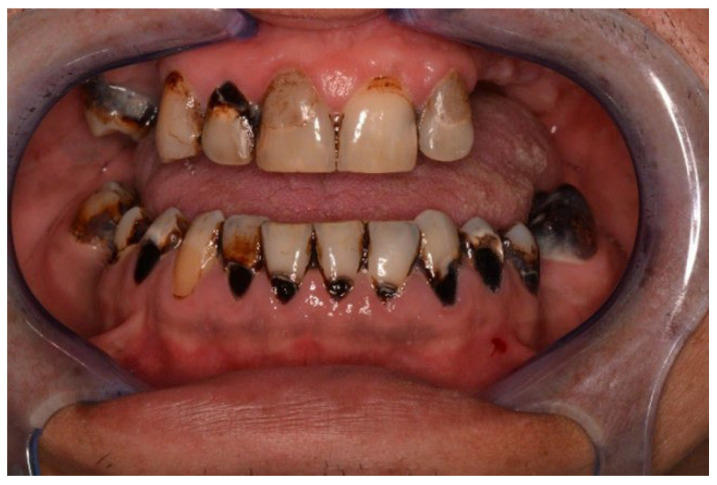
38-year-old man, 4 weeks to alloHSCT.

The high prevalence and intensity of caries were also alarming given the brief time available for dental treatment. The number of infectious foci requiring elimination was high, often necessitating multiple visits (Figure 6). In the study by Durey et al., the average time remaining before HSCT was longer (M = 31.5 days; SD = 18.8), with a similar average number of visits (M = 2) [17]. However, those authors also included procedures such as scaling and prosthetic restoration [17]. As noted in our research, factors limiting dental treatment time included other required tests or specialized consultations (e.g., ENT—Ear, Nose, Throat; gynecology) and instances where visit duration had to be shortened due to the patient’s poor condition.

Analyzing the reasons for the high prevalence and intensity of dental caries in patients that qualified for HSCT, we found the influence of some important factors. The caries severity index significantly increased with the API value. Notably, 33% of HSCT candidates brushed their teeth only once a day or not at all. While this reflects trends in the general Polish population, it is worrisome, as it indicates an increased risk of rapid caries development and post-transplantation complications (Figure 3 and Figure 5). Similar data was not found in the available literature [36].

**Figure 7:**
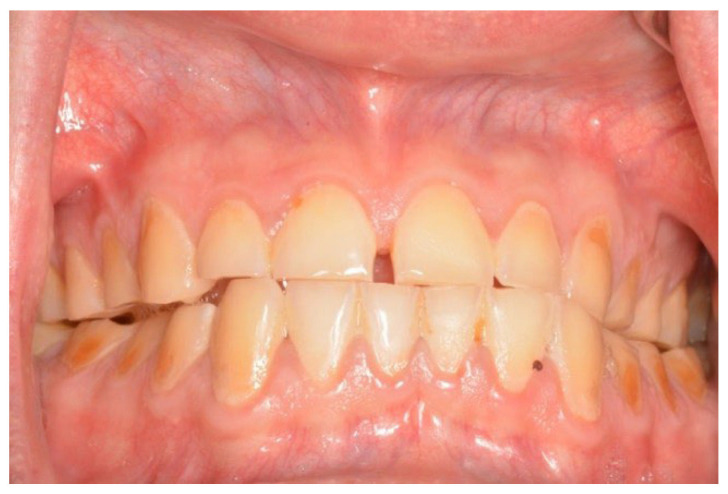
32-year-old man, frequent consumption of sweet and low-pH beverages between meals, driven by chemotherapy-induced taste disturbances.

Caries progression could also have been influenced by prolonged reduced salivary secretion. This could have been a complication of previous anticancer therapy (chemotherapy or radiotherapy) [37]. As a sequel to chemotherapy, hyposalivation leads to altered salivary composition, including reduced buffering capacity, which acidifies the oral environment and damages dental tissues [38]. Low pH, poor hygiene, and chemotherapy-associated immunodeficiency also promote the proliferation of cariogenic bacteria and oral dysbiosis [39]. Another significant risk factor was the frequent consumption of simple sugars between meals, potentially driven by taste disturbances induced by chemotherapy (Figure 7) [40].

The current study has its limitations. Resource constraints restricted the scope to three Polish transplantation centers in economically well-developed provinces; therefore, higher caries prevalence and severity might be expected in other regions.

Furthermore, the self-administered questionnaire is subject to bias and the patients’ emotional or physical state.

The authors acknowledge that the lack of objective salivary flow rate measurement is a limitation. Xerostomia is the subjective sensation of dry mouth, which may occur without salivary gland hypofunction. Currently, salivary flow rate assessment is the most reliable method to confirm hyposalivation. However, in this specific patient group, such measurements were often impossible due to:

- Often significant weakness and fatigue (the oral examination was already time-consuming).
- Frequent gag reflexes during saliva collection or paraffin chewing.
- Complete absence or scant volume of thick, difficult-to-expectorate secretions.

Consequently, reduced salivary flow was assessed indirectly via clinical examination and a questionnaire based on Sreebny et al., where the question “Does your mouth usually feel dry?” demonstrated high sensitivity (93%) for hyposalivation [20].

The questionnaire was considered a key element of the study. Although the sensation of dry mouth appeared to be a statistically insignificant predictor of hyposalivation in our research, we observed that the subjective sensation of oral dryness—regardless of the presence or absence of objective hyposalivation—significantly influenced patients’ dietary behaviors. For example, patients with xerostomia were often reluctant to drink plain water, frequently replacing it with sweetened beverages. Taste disorders, mentioned previously, were of additional significance, as patients often alleviated these symptoms by adding sugar to their drinks. Consequently, this contributed to the development of caries, dental erosion, and dentin hypersensitivity (Figure 7). Furthermore, patients with a severe sensation of dry mouth (score > 5/10) often reduced their daily oral hygiene routines due to unpleasant mucosal symptoms, such as stinging or pain caused by toothpaste or the physical contact of the toothbrush.

In the clinical examination, particular attention was paid to signs indicating reduced or absent salivary secretion. These symptoms were categorized into two groups:

- Insufficient mucosal lubrication, which could be caused, for example, by a temporary reduction in saliva associated with the use of medicines, like antidepressants (Grade 2 on a three-point scale).
- Mucosal dryness, indicating a significant deficiency or complete lack of saliva (Grade 3 on a three-point scale) [23].

Additionally, other factors potentially affecting caries development—such as the patients’ general health, the duration of the underlying disease, and the type of treatment received—should be considered.

It is also important to acknowledge the limitations of commonly used visual–tactile indices for detecting and assessing caries-related conditions. In this study, the WHO DMF index was used to assess the prevalence and consequences of caries [19]. Despite its limitations (e.g., recording only cavitated lesions), this index was chosen due to its global acceptance and the ability to compare our results with other findings, particularly those from the national oral health monitoring project in Poland [36]. Nevertheless, clinicians must always account for the specific clinical presentation of caries in patients who have undergone oncological therapy, which often includes rapid progression, atypical locations (cervical or incisal surfaces), and subsurface lesions [41].

## 5. Conclusions

A high prevalence of active dental caries, high caries intensity, and a low caries treatment index were observed among adult patients that qualified for HSCT in the Polish study population. Insufficient oral hygiene, improper diet, indirect and subjective symptoms of reduced salivary flow should be considered key local factors indicating a high risk for caries in patients prior to transplantation. In this context, the role of the dental hygienist deserves special emphasis. Unfortunately, the implementation of individualized caries prevention plans for oncological patients remains underestimated in Poland and the pre-oncologic dental treatment of patients with hematopoietic disorders does not yet meet the MASCC/ISOO standards.

A disconcerting finding is that the time available for dental preparation prior to HSCT is often insufficient. This study suggests that the primary challenge is a lack of coordination between oncological and dental care in Poland. Patients are rarely referred to a dentist by oncologists before beginning cancer therapy; consequently, those scheduled for transplantation often seek dental consultation too late.

Our study highlights the urgent need to intensify systemic efforts to provide specialized dental care for HSCT candidates, ensuring optimal oral health before the onset of myelosuppression. Identifying dental caries foci and assessing predisposing factors will enable the establishment and early implementation of individualized prevention and treatment protocols. Such measures will contribute to mitigating both short- and long-term risks of local and systemic caries-related complications in post-HSCT patients.

## Figures and Tables

**Table 1:** Diagnosis before HSCT.

Disease	*N*	%
Acute myeloid leukemia (AML)	105	34.76
Acute lymphoblastic leukemia (ALL)	38	12.58
Chronic myeloid leukemia (CML)	21	6.95
Chronic lymphocytic leukemia (CLL)	1	0.33
Myelodysplastic syndrome (MDS)	28	9.27
Myelofibrosis	9	2.98
Hodgkin’s lymphoma	24	7.94
Non-Hodgkin’s lymphoma	24	7.94
Myeloma	29	9.60
Thrombocytopenia	3	0.99
Bone marrow aplasia	11	3.64
Other	9	2.98
Total	302	100

*N*—the number of patients.

**Table 5:** The predictors of active caries in patients before HSCT.

	B	SE	Wald	*p*	Exp(B)	95% CI	
						LL	UL
Oral mucosal hydration	−0.48	0.25	3.89	0.049	0.62	0.38	0.997
API	0.02	0.01	16.985	<0.001	1.02	1.01	1.03
Constant	0.22	0.50	0.20	0.658	1.25		

B—unstandardized regression coefficient; SE—Standard Error; Exp(B)—Odds Ratio; 95% CI—Confidence Interval; LL—Lower Limit; UL—Upper Limit.

**Table 6:** Teeth brushing frequency and DMFT, SiC and AP Indices (*N* = 302).

Index	Sporadically or Not at All (*N* = 27)		Once a Day (*N* = 64)		Two Times a Day (*N* = 199)		More Than 2 Times Daily (*N* = 12)			
	M	SD	M	SD	M	SD	M	SD	H	*p*
DMFT	24	6.1	21.6	7.6	17.2	7.9	20.7	6.9	29.7	<0.001
SiC	28.5	2.8	27.4	2.4	27.1	2.3	27	3.6	2.9	0.408
API	85.4	18.7	70.6	17.5	48	22.3	45	33.3	81	<0.001

DMFT Index = Decayed (DT) + Missing (MT) + Filled (FT) Teeth Index; SiC—Significant Caries Index; API—Approximal Plaque Index; M—mean; SD—standard deviation.

## Data Availability

The data that support the findings of this study are not openly available due to reasons of sensitivity and are available from the corresponding author upon reasonable request. Data are located in controlled access data storage at Warsaw Medical University, Poland.
